# Regulated Control of Melanin-Concentrating Hormone Receptor 1 through Posttranslational Modifications

**DOI:** 10.3389/fendo.2013.00154

**Published:** 2013-10-21

**Authors:** Yumiko Saito, Akie Hamamoto, Yuki Kobayashi

**Affiliations:** ^1^Graduate School of Integrated Arts and Sciences, Hiroshima University, Hiroshima, Japan

**Keywords:** melanin-concentrating hormone, structure-function relationship, glycosylation, phosphorylation, G-protein

## Abstract

Melanin-concentrating hormone (MCH) is a hypothalamic neuropeptide that plays an important role in feeding behavior. It activates two G-protein-coupled receptors, MCHR1 and MCHR2, of which MCHR1 is the primary regulator of food intake and energy homeostasis in rodents. In mammalian cells transfected with MCHR1, MCH is able to activate multiple signaling pathways including calcium mobilization, extracellular signal-regulated kinase activation, and inhibition of cyclic AMP generation through Gi/o- and Gq-coupled pathways. Further evidence suggests that MCHR1 is regulated through posttranslational modifications, which control its intracellular localization and provide appropriate cellular responses involving G-protein signaling. This review summarizes the current data on the control of MCHR1 function through glycosylation and phosphorylation, as related to cell function. Especially, a series of mutagenesis study highlights the importance of complete glycosylation of MCHR1 for efficient trafficking to the plasma membrane.

## Introduction

The G-protein-coupled receptor (GPCR) gene family is one of the largest families in the mammalian genome. GPCRs are typical heptahelical receptors composed of an extracellular N-terminus, an intracellular C-terminus, and seven transmembrane bundles connected by three intracellular loops and three extracellular loops. Activation of GPCRs induces second messenger responses that change the biochemical properties of the recipient cell and can modulate not only its electrophysiological responsiveness, but also its transcriptional activity. Thus, the diverse signaling and wide array of functions have allowed GPCRs to be employed in the physiological regulation of nearly all biological functions. These features coupled with ligands that are chemically highly specific for the receptors have resulted in the extensive utilization of GPCR-targeted drug design. Furthermore, the dynamic posttranslational modifications may provide tissue-specific functions, since distinct cellular environments or agonists can mediate different effects on receptor signaling or regulation of a number of GPCRs (1–5). Consequently, these regulatory processes may hold the keys to alternative targets for GPCR research. In this article, we focus solely on the posttranslational modification of one GPCR, melanin-concentrating hormone (MCH) receptor, which is one of the potential targets for obesity research.

## Role of MCH in Food Intake

Melanin-concentrating hormone was originally discovered as a 17-amino-acid neuropeptide in the chum salmon pituitary (6). MCH is secreted from the pituitary into the circulation, and induces paling of the skin in teleost fish. Mammalian MCH was subsequently identified as a 19-amino-acid peptide in the rat hypothalamus (7). Although the peptide structures are highly conserved between fish and mammals, no direct effects of MCH on skin pigmentation in mammals are demonstrable. Mammalian MCH is predominantly synthesized in the brain, especially in the lateral hypothalamus (LH) and zona incerta with projections to numerous areas in the brain (8). The LH is classically known as the “hunger center,” since lesions in this area produce anorexia and stimulation of the area leads to overeating. The important role of MCH in feeding was supported by a study showing that the *MCH* gene is overexpressed upon fasting and in *ob*/*ob* leptin-deficient mice (9). Moreover, direct intracerebroventricular administration of MCH increases food intake in rats, suggesting that MCH is an orexigenic peptide (9), while chronic infusion of MCH or MCH analogs significantly increases food intake, body weight, white adipose tissue mass, and liver mass in mice fed a moderately high-fat diet *ad libitum* (10, 11). Further evidence of the significance of MCH in feeding came from studying the effects of altering the MCH levels using knockout and overexpression techniques (12–14). It has shown that ablation of functional MCH results in a lean phenotype, increased energy expenditure, and resistance to diet-induced obesity. Such phenotypes are not observed for many other neuropeptides, suggesting a crucial role for MCH in feeding behavior. On the basis of these data, MCH appears to be a critical effector of feeding behavior and energy balance.

## MCH Acts through GPCRs

Despite the discovery of the MCH peptide, the site of its biological action remained obscure until 1999. At that time, five independent groups, including us, identified that the MCH receptor (MCHR1) was SLC-1/GPR24, an orphan GPCR, by applying orphan receptor strategies and reverse pharmacology (15–19). MCHR1 belongs to the γ-group of rhodopsin family class A GPCRs (20), and shows 40% homology with the somatostatin receptor as its closest neighbor. High expression of MCHR1 mRNA in rats is detected in most anatomical areas implicated in the control of olfaction, such as the olfactory nerve layer, olfactory nucleus, and tubercle (21). Strong labeling is also detected in several limbic structures, such as the hippocampal formation, septum, and amygdala, all of which are implicated in the regulation of stress and emotional processes. Furthermore, MCHR1 is abundantly expressed in the nucleus accumbens shell, where it may play roles in the regulation of motivation and reward. In recombinant cell lines, MCH binds to MCHR1 with affinities of ∼1 nM, and couples to Gi, Go, and Gq proteins (15, 16, 22). Thus, activation of MCHR1 leads to increases in intracellular calcium mobilization via both Gi/o- and Gq-coupled pathways and to decreased cyclic AMP (cAMP) levels via the Gi/o-coupled pathway. Further analyses of MCHR1 signaling in recombinant cell lines and hippocampal brain slices demonstrated that activation of MCHR1 also leads to extracellular signal-regulated kinase (ERK) phosphorylation (22, 23). Recently, accumulating evidence has supported the notion that receptor-binding partners regulate the magnitude, duration, and spatial components of GPCR signaling. MCHR1-binding proteins have also been detected and described. Periplakin and neurochondrin, which interact with the proximal C-terminus of MCHR1, reduce the capacity to initiate calcium mobilization (24, 25). Furthermore, RGS8, one of the GTPase-activating proteins for Gα subunits, was identified as a negative regulator. Arg253 and Arg256 at the distal end of the third cytoplasmic loop were found to comprise a structurally important site for the functional interaction with RGS8 (26). Clarification of the physiological consequences of these proteins that interact with the MCHR1 system will be achieved by assessing their coexpressions in the nervous system.

A second MCH receptor (MCHR2) was subsequently identified by six groups using human genomic sequence searches (27). It shares 38% amino acid identity with MCHR1 and binds to MCH with high affinity (28). The distribution of MCHR2 in humans is relatively limited, in that it is expressed in the cerebral cortex, amygdala, and hippocampus, but not in the hypothalamus (29). In contrast to human MCHR1, human MCHR2 only couples to Gq protein, and the signaling is not sensitive to pertussis toxin. Of note, MCHR2 was found to be a pseudogene in rodent species, but is functional in dogs, ferrets, rhesus monkeys, and humans (30). The physiological importance of MCHR2 remains unknown owing to the lack of available animal models. Later studies identified three MCH receptor sequences in zebrafish and two receptor sequences from fugu, barfin flounder, and goldfish in whole-genome datasets (31–33). Moreover, based on predictions from a preliminary genome assembly of *Xenopus tropicalis*, information for four MCH receptors has been obtained (34). Phylogenetic analyses of these receptors suggest that an initial duplication of the MCH receptor occurred early in evolution, giving rise to MCHR1 and MCHR2. MCH receptors are only found in vertebrates (35). Therefore, the characterization of MCH receptors from birds and reptiles may serve as a valuable reference to elucidate the role of the MCH system during evolution.

The gene for prepro MCH encodes two additional peptides, NEI and NGE of unknown function. MCHR1 is the sole receptor expressed in rodents, MCHR1 knockout mice may provide confirmation of the role of MCH in energy homeostasis. To date, MCHR1 knockout mice have been reported to exhibit an obesity-prone phenotype (36, 37). In contrast to MCH knockout mice, MCHR1 knockout mice remained lean on a regular chow diet. On a high-fat diet, the mice gained less weight, characterized by hyperphagia, hyperactivity, and hypermetabolism. To better understand the role of MCH-MCHR1 system in energy homeostasis, several structurally distinct small molecule antagonists for the MCHR1 have been synthesized and tested in cell-based assays for their selectivity and for *in vivo* potency in the rodent. Comprehensive review of desired selectivity and efficacy of MCHR1 antagonist *in vivo* should consult some recent reviews (38, 39). The majority of studies indicated that the MCHR1 antagonists are effective in different models of obesity in a variety of different rodent strains, due to inhibition of food intake and/or energy expenditure (40, 41). Overall, both rodent genetic studies and rodent pharmacologic studies on MCHR1 have confirmed the importance of the MCH-MCHR1 system for modulating energy homeostasis. With the broad distribution of MCH fibers and MCHR1 in the rodent brain, the physiological function of the MCH system is not only restricted to feeding behavior. In accordance with these observations, multiple rodent models have suggested functional implications of the MCH-MCHR1 system in sleep, emotion, and reward effects of psychostimulants (42–45). In peripheral tissues, the MCH-MCHR1 system has been shown to play important roles in pancreatic islet function and intestinal inflammation (46, 47).

## Posttranslational Control of MCH Receptors

Posttranslational modifications are considered to be the primary regulatory mechanisms of virtually all GPCRs. Since most GPCRs are naturally expressed at low levels, except for rhodopsin, *in vitro* eukaryotic heterologous expression systems are often employed for their biochemical characterization, including the complex posttranslational modifications.

The most well-understood posttranslational modifications include palmitoylation, glycosylation, and phosphorylation. Most GPCRs are posttranslationally modified with one or more palmitic acids, a 16-carbon saturated fatty acid, covalently bound to cysteine(s) localized in the C-terminal cytoplasmic tail. The insertion of palmitate into the cytoplasmic leaflet of the plasma membrane can create a fourth loop named helix 8, thereby profoundly affecting the GPCR structure and consequently the interactions with intracellular partner proteins. Although a putative palmitoylation site has not been identified in mammalian MCHR1 (48), the existence of helix 8 was predicted by a computational analysis and its functional importance in the signaling pathway has been experimentally confirmed in HEK293T cells (49, 50).

**Figure 1:**
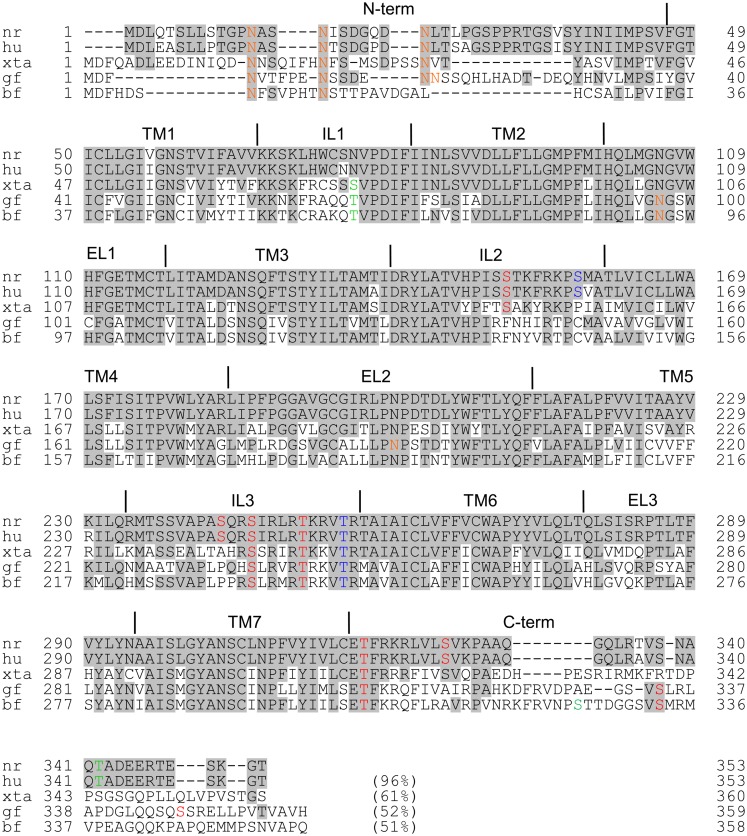
**Amino acid comparisons of Norway rat MCHR1 and its mammalian, amphibian, and fish orthologs**. The accession numbers are as follows: Norway rat (nr), NP_113946; human (hu), NP_005288; *Xenopus tropicalis* 1a (xta), NP_001072243; goldfish (gf), BAH70338; barfin flounder (bf), BAF49517. Common amino acids with rat MCHR1 are shaded. Colored amino acids show individual motifs: orange, N-glycosylation site; green, casein kinase 2 phosphorylation site; red, protein kinase C phosphorylation site; blue, cAMP-dependent protein kinase phosphorylation site. The numbers in parentheses show the sequence identities with the rat sequence.

N-linked glycosylation is one of the most common forms of posttranslational modification. The consensus sequences for N-linked glycosylation, Asn-X-Thr and Asn-X-Ser, in which oligosaccharides can bind to the asparagine residues, are found in many GPCRs and are shared by almost all eukaryotic cells, including yeast cells. The significance of these asparagine residues has been demonstrated in various receptors, and they seem to play several important roles including receptor folding, trafficking, and stability, thereby allowing fine-tuning of the receptor function. When Flag-tagged rat MCHR1 is expressed in HEK293 cells, several characteristic immunoreactive bands of ∼35–65 kDa are detected by western blotting (51). These molecular weights are higher than those predicted from MCHR1 cDNA sequences, suggesting glycosylated forms of MCHR1. This notion is supported by the fact that the N-terminal domain of rat MCHR1 includes three N-glycosylation sites at Asn13, Asn16, and Asn23 (48), all of which are conserved among the goldfish, *Xenopus*, mouse, and human orthologs (Figure 1). We proved the presence of oligosaccharides in MCHR1 by enzymatic treatment. Furthermore, single or multiple mutations of Asn13, Asn16, and Asn23 to Gln (N23Q, N13Q/N16Q, N13Q/N23Q, N16Q/N23Q, and N13Q/N16Q/N23Q) caused pronounced reductions in the levels of glycosylation of the receptor protein, and impaired the receptor expression at the cell surface. In particular, the N-linked glycosylation at Asn23 is necessary for the cell surface expression and signal transduction (51, 52). Mutation of residues other than the N-glycosylation sites also caused incomplete glycosylation of MCHR1, and impaired receptor trafficking and signaling. MCHR1 belongs to the rhodopsin family of GPCRs, and the Glu/Asp3.49-Arg3.50-Tyr3.51 (E/DRY) sequence is a highly conserved motif in this family. It was found that substitution of Asp140 or Tyr142 to Ala (D140A and Y142A, respectively) resulted in non-functional receptors without changing the high affinity constant values in HEK293 cells (53). The cell surface expression levels of both mutants were about 40% of the wild-type MCHR1 level, with pronounced decreases in the 65-kDa immunoreactive band (highly glycosylated form). Different types of site-directed mutants that exhibited much less glycosylation were found for highly conserved Pro residues at transmembrane regions 2, 4, 5, 6, and 7 (P97A, P177A, P220A, P271A, and P308A, respectively) (Figure 1). These mutations all produced complete loss of signaling with lack of the 65-kDa immunoreactive band in HEK293 cells (50). The importance of proper glycosylation for receptor trafficking and signaling was extensively analyzed using substitution of Thr255 to Ala (T255A), which is located at the junction of intracellular loop 3 and transmembrane region 6 (52). T255A was largely retained in the endoplasmic reticulum (ER) and associated with the chaperon protein calnexin in HEK293 cells. This arose through receptor misfolding, which prevented some N-glycosylation. Interestingly, addition of a small molecule MCHR1 antagonist to T255A- and N13Q/N16Q/N23Q-expressing cells led to functional receptors becoming fully glycosylated at similar extents to wild-type MCHR1. Thus, complete glycosylation of MCHR1 is necessary to allow its efficient trafficking through the ER and Golgi to the plasma membrane.

Ligand-stimulated GPCR activation, desensitization, internalization, and recycling occur in a controlled cyclical process. Since the magnitude and duration of ligand-induced GPCR responses are linked to the balance between signal generation and signal termination, the endocytic pathway that starts with internalization tightly controls the activity of GPCRs. The process of endocytosis is promoted by agonist-induced phosphorylation of receptors. Upon GPCR activation, several sites in GPCRs are immediately phosphorylated by G-protein receptor kinases and other protein kinases. The receptor phosphorylation and subsequent binding of β-arrestin prevent consequent interactions of the receptors with G-proteins, thereby effectively terminating the G-protein-mediated signaling and initiating the endocytic process. The endocytic activity of arrestin is also subject to dynamic regulation by dephosphorylation and ubiquitination. GPCR phosphorylation usually occurs predominantly on Ser and Thr residues within the C-terminal receptor tail and third intracellular loop. In the intracellular loop of rat MCHR1, there are nine predicted phosphorylation sites, comprising two for protein kinase A (Arg/Lys–Arg/Lys–X–Ser/Thr: S158 and T255), six for protein kinase C (Ser/Thr–X–Arg/Lys: S151, S243, S246, T251, T317, and S325), and one for protein kinase casein kinase 2 (Ser/Thr–X–X–Asp/Glu: T342) (Figure 1). It has been described that the Ser and Thr residues are phosphorylated in agonist-independent manners in some GPCRs (54). However, to our knowledge, no previous studies have identified whether MCHR1 is phosphorylated under basal conditions or in response to MCH using a [^32^P]orthophosphate metabolic pre-labeling approach. On the other hand, the results of a mutational study suggested the importance of several predicted phosphorylation sites in MCHR1 for the receptor function. A triple substituted mutant in the C-terminus, T317A/S325A/T342A, has no effects on the signal transduction in calcium mobilization, but significantly prevents MCH-induced receptor internalization through protein kinase C and β-arrestin 2-dependent processes (55). Since S246, T251, and T255 in the third intracellular loop are all conserved in MCHR1 derived from goldfish, *Xenopus*, mice, and humans (Figure 1), they may play a critical role in receptor function via phosphorylation. The T255A mutation produces a non-functional receptor as described above (52), while the importance of S245 and T251 remains unclear. Thus far, we have preliminary data that point mutations at these residues produce no significant changes in either the cellular localization or calcium mobilization (Honda and Hamamoto, unpublished data). It has been reported that the generation of phosphosite-specific antibodies accompanied by site-directed mutagenesis and chemical kinase inhibitors can provide more direct evidence for phosphorylation in GPCR-expressing cells (2, 3). Since rat MCHR1 possesses nine potential phosphorylation sites, a series of phosphate acceptor site-specific antibodies are potentially obtainable to reveal the coordinated biochemical mechanism involving sequential and hierarchical multisite phosphorylation of the receptor, similar to somatostatin or μ opioid receptors (4, 56).

## Future Directions

The MCH-MCHR1 system has integral roles in many cellular events, and represents an important therapeutic target. For example, it may be targeted in strategies for developing treatments against obesity and mood disorders, and possibly also for inflammatory diseases. Therefore, the regulation of the MCH system by posttranslational modifications remains an emerging area at the nexus of endocrinology with important implications for drug development. However, our understanding of the regulation of MCHR1 signaling by glycosylation and phosphorylation is limited (Table 1). At present, several methodological advances could facilitate analyses of GPCR posttranslational modifications. In particular, significant improvements have been achieved in mass spectrometry (MS) and related procedures such as tandem MS or chemical microsequencing, such that MS is becoming a major tool for analyzing posttranslational modifications. Since purification of hydrophobic membrane proteins has been enhanced by the utilization of specific detergents and several enrichment procedures, MS analyses could have broad utility, such as direct identification of glycosylated or phosphorylated residues, even for low amounts in GPCRs (1, 5, 57, 58). Moreover, it has been shown that distinct cellular environments can mediate different glycosylation patterns in rhodopsin and serotonin receptor type 4 (5). For example, rhodopsin is heavily and heterogeneously glycosylated when expressed in HEK293 and COS-1 cells, but shows a sparser and more homogeneous glycosylation pattern in its native retinal tissue. Thus, these features remind us of the significant importance of studying both heterologous and natural expression systems, and offer a comprehensive view of the posttranslational modifications in MCHR1.

**Table 1:** **Posttranslational modifications with cellular function in mammal MCHR1**.

Region		Mutant receptor	Impact on MCHR1 in HEK293 cells	Reference
Extracellular N-terminus		N23Q^a^		
		N13Q/N16Q^a^	⇓ Mature glycosylation	
		N13Q/N23Q^a^	⇓ Cell surface expression	(51, 52)
		N16Q/N23Q^a^	⇓ Ca^2+^ mobilization	
		N13Q/N16Q/N23Q^a^		

Intracellular loop 2		D140A	⇓ Mature glycosylation	(53)
		Y142A	⇓ Cell surface expression
			Loss of function (Ca^2+^ mobilization, cAMP inhibition, ERK activation)

Transmembrane region	2	P97A		
	4	P177A		
			⇓ Mature glycosylation
	5	P220A		
	6	P271A	
			Loss of function (Ca^2+^ mobilization)
	7	P308A		(50)

Junction of intracellular loop 3 and transmembrane region 6		T255A^b^	⇓ Mature glycosylation	(52)
			⇓ Cell surface expression (retained in the ER)	
			Loss of function (Ca^2+^ mobilization)	

Intracellular C-terminus		T317A/S325A/T342A^b^	**→** Ca^2+^ mobilization	(55)
			⇓ Receptor internalization (protein kinase C and β-arrestin 2-dependent)	

^a^The consensus sequences for N-linked glycosylation.

^b^Predicted phosphorylation sites.

## Conflict of Interest Statement

The authors declare that the research was conducted in the absence of any commercial or financial relationships that could be construed as a potential conflict of interest.
